# Carbon Black Nanoparticles Promote Endothelial Activation and Lipid Accumulation in Macrophages Independently of Intracellular ROS Production

**DOI:** 10.1371/journal.pone.0106711

**Published:** 2014-09-03

**Authors:** Yi Cao, Martin Roursgaard, Pernille Høgh Danielsen, Peter Møller, Steffen Loft

**Affiliations:** Section of Environmental Health, Department of Public Health, University of Copenhagen, Copenhagen, Denmark; Fundação Oswaldo Cruz, Brazil

## Abstract

Exposure to nanoparticles (NPs) may cause vascular effects including endothelial dysfunction and foam cell formation, with oxidative stress and inflammation as supposed central mechanisms. We investigated oxidative stress, endothelial dysfunction and lipid accumulation caused by nano-sized carbon black (CB) exposure in cultured human umbilical vein endothelial cells (HUVECs), THP-1 (monocytes) and THP-1 derived macrophages (THP-1a). The proliferation of HUVECs or co-cultures of HUVECs and THP-1 cells were unaffected by CB exposure, whereas there was increased cytotoxicity, assessed by the LDH and WST-1 assays, especially in THP-1 and THP-1a cells. The CB exposure decreased the glutathione (GSH) content in THP-1 and THP-1a cells, whereas GSH was increased in HUVECs. The reactive oxygen species (ROS) production was increased in all cell types after CB exposure. A reduction of the intracellular GSH concentration by buthionine sulfoximine (BSO) pre-treatment further increased the CB-induced ROS production in THP-1 cells and HUVECs. The expression of adhesion molecules ICAM-1 and VCAM-1, but not adhesion of THP-1 to HUVECs or culture dishes, was elevated by CB exposure, whereas these effects were unaffected by BSO pre-treatment. qRT-PCR showed increased *VCAM1* expression, but no change in *GCLM* and *HMOX1* expression in CB-exposed HUVECs. Pre-exposure to CB induced lipid accumulation in THP-1a cells, which was not affected by the presence of the antioxidant N-acetylcysteine. In addition, the concentrations of CB to induce lipid accumulation were lower than the concentrations to promote intracellular ROS production in THP-1a cells. In conclusion, exposure to nano-sized CB induced endothelial dysfunction and foam cell formation, which was not dependent on intracellular ROS production.

## Introduction

Exposure to nanoparticles (NPs) has been suggested to cause vascular health effects with oxidative stress and inflammation as central mechanisms [1]. The NP-mediated vascular effects include expression of endothelial cell adhesion molecules such as intercellular adhesion molecule 1 (ICAM-1) and vascular cell adhesion molecule 1 (VCAM-1), vasomotor dysfunction and accelerated progression of atherosclerosis [1]. The expression of ICAM-1 and VCAM-1 promotes the firm adhesion of monocytes onto the endothelium and the monocytes can subsequently differentiate into macrophages, migrate to the intima and transform to foam cells [2]. It has been shown that exposure of endothelial cells to NPs promotes the expression of ICAM-1 and VCAM-1 as well as adhesion of monocytes onto the endothelial cells [3], [4]. Furthermore, it has also been shown that NP exposure induces intracellular lipid accumulation [5]–[7]. The process of endothelial activation might not require oxidative stress, as suggested by increased adhesion molecule expression by NP exposure in a manner not associated with generation of ROS [8], [9]. In addition, it has been shown that addition of the antioxidant ascorbic acid to the cell culture medium did not alleviate particle-induced ICAM-1 and VCAM-1 expression on human umbilical vein endothelial cells (HUVECs) [10]. On the other hand, NP induced lipid accumulation in rat cells was inhibited by pre-treatment with the antioxidant N-acetylcysteine (NAC) [11].

We hypothesized that oxidatively stressed endothelial cells would be more readily activated and interact more strongly with monocytes or macrophages, and that oxidative stress could further promote the lipid accumulation in macrophages by exposure to NPs. To this end we investigated the effect of exposure to nano-sized carbon black (CB) on the activation of endothelial cells by ICAM-1 and VCAM-1 expression on HUVECs and adhesion of THP-1 monocytes onto HUVECs as well as lipid accumulation in THP-1 macrophages. We used nano-sized CB because it generates high levels of intracellular ROS [12]. In addition, we have previously shown that HUVECs express increased levels of ICAM-1 and VCAM-1 after exposure to nano-sized CB [9], [10], [13]. CB is widely used as black pigment in rubber, paints and inks as well as being a widely used type of particle in toxicological studies including studies on ROS production [14], endothelial-dependent vasomotor function [15] and atherosclerosis [16]. The intracellular ROS generation and GSH concentration were used as markers of oxidative stress, whereas the mRNA expression of adhesion molecule *VCAM-1* as well as the oxidative stress response genes in the NRF-2 signaling pathway, glutamate-cysteine ligase, modifier subunit (*GCLM*), the rate limiting enzyme in GSH synthesis, and heme oxygenase 1 (*HMOX1*), one of the essential enzymes in heme catabolism [17], was assessed in HUVECs by qRT-PCR.

## Materials and Methods

### Cell lines

The HUVECs and culture medium were purchased from Cell Applications (San Diego, CA, USA). The cells were cultured in Endothelial Cell Growth Medium Kit with 2% serum at 37°C in an incubator with 5% CO_2_. The medium was changed 24–36 h after seeding and the cells were cultured until they were 90% confluent. The cells were used between passages 2–5 because they maintain their morphologic and phenotypic characteristics within these passages [9], [13]. The THP-1 monocytes were obtained from the American Type Culture Collection (Manassas, VA, USA) and was cultured in RPMI with 10% serum as previously described [18]. The THP-1 cells were differentiated into adherent macrophages (denoted THP-1a) by treatment with 10 ng/ml phorbol 12-myristate 13-acetate (PMA, Sigma, St. Louis, MO, USA) overnight [19]. The THP-1a cells attach to the surface of the culture flasks, whereas THP-1 cells stay in suspension and were removed with the supernatant.

### Particles

CB particles (Printex 90) were obtained from Evonik Industries, Frankfurt, Germany (primary particle size 14 nm; surface area 300 m^2^/g). Printex 90 is an extensively studied model NP and has been characterized elsewhere [20], [21]. The mean size of the particles suspended in media was 85±38 nm [13]. The uptake of CB in cells has been confirmed by transmission electron micrograph (TEM) [13]. A stock solution of CB was prepared by sonicating a 1 mg/ml suspension of particles in double distilled water (Sigma-Aldrich), using a Branson Sonifier S-450D (Branson Ultrasonics Corp., Danbury, CT, USA) equipped with a disrupter horn (Model number: 101-147-037) before each experiment. The suspension was sonicated for 8 min with alternating 10 s pulses and 10 s pauses at amplitude of 10% and continuously cooling on ice to avoid sample heating and evaporation [13]. After sonication, the suspension was diluted in cell culture medium and used only freshly. For all the experiments except real-time RT-PCR, cells were incubated with 200 µl CB solutions in 96-wells plates (growth area 0.32 cm^2^). The concentrations of CB were 0, 2.5, 12.5, 25, and 100 µg/ml, which were equal to the concentrations of 0, 1.6, 7.8, 15.6, 62.5 µg/cm^2^. Our previous studies showed increased levels of ICAM-1 and VCAM-1 in HUVECs after exposure to these concentrations of CB [9], [10], [13]. As we observed increased lipid accumulation in THP-1a cells by CB as low as 2.5 µg/ml (see in results section), we also included lower concentrations of CB of 0.25, 2.5, 25, 250, 2500 ng/ml, which were equal to the concentrations of 0.16, 1.6, 15.6, 156.3, 1562.5 ng/cm^2^. For real-time RT-PCR, cells were incubated with 5 ml CB solutions in 6-wells plates.

### Cytotoxicity

The cytotoxicity was measured with the WST-1 or lactate dehydrogenase (LDH) assays (Roche Diagnostics GmbH, Mannheim, Germany) according to the manufacturer's instructions. The WST-1 assay is regarded to reflect mitochondrial succinate dehydrogenase activity, although it should not be interpreted as a specific measurement for this enzyme. The LDH assay is considered to reflect leakage of this cytosolic enzyme through the cell membrane to the extracellular fluid.

Briefly, 5×10^4^ THP-1, 5×10^4^ THP-1a or 2×10^4^ HUVECs were seeded in 96-wells plates and incubated with CB for 24 h. Then, the supernatant was collected for LDH assay and cells were rinsed once for WST-1 assay. The cells were incubated with 100 µl fresh medium containing 10% WST-1 reagent for 2 h and the absorbance was measured at 450 nm with 630 nm as reference by an ELISA reader (Labsystems, Multiskan Ascent). For LDH assay, 100 µl supernatant was incubated with 100 µl LDH reaction buffer provided in the kit for 1 h and the absorbance was measured at 490 nm with 630 nm as reference using an ELISA reader. We have previously shown that the CB particles do not interact with LDH enzyme activity [13], [22].

Cell viability was further assessed in THP-1 cells by trypan blue exclusion. After exposure of 5×10^4^ THP-1 cells to 100 µg/ml CB, 40 µl cell suspension was mixed with 40 µl 0.4% trypan blue. The non-colored viable cells and blue-stained non-viable cells were counted in a hemacytometer.

### Cell proliferation assay

The proliferation of THP-1 cells and HUVECs was measured with the BrdU cell proliferation assay kit (Roche Diagnostics GmbH, Mannheim, Germany) according to the manufacturer's instructions. Briefly, 2×10^4^ THP-1, 5×10^3^ HUVECs or a mixture of THP-1 or THP-1a (1×10^4^) with HUVECs (2.5×10^3^) were exposed to CB suspension in the presence of BrdU for 24 h. After exposure, the medium were removed and cells were fixed for 30 min in fix solution, and then the BrdU-labelled DNA was bind to antibody for 90 min in antibody solution. After washing three times, the substrate solution was added to detect the immune complexes. The reaction was stopped with 50 µl 2 M HCl, and the absorbance was measured at 450 nm with 690 nm as reference. To confirm the proliferation results of THP-1 cell, 5×10^4^/well of THP-1 cells were exposed to 100 µg/ml CB for 24 h, and the cell number was counted using a CASY Technology Cell counter (Scharfe System, Stuttgart, Germany). The proliferation of HUVECs was also measured after 24 h exposure to 10 ng/ml vascular endothelial growth factor (VEGF, Gibco, Camarillo, CA, USA) to assess the maximal proliferation capacity or 100 ng/ml tumor necrosis factor (TNF, Gibco, Carlsbad, CA, USA) because it is used as positive control for the expression of cell adhesion molecules (see below). Moreover, we assessed the proliferation of HUVECs after pre-treatment with the Ca^2+^ chelator BAPTA-AM (10 µM; Sigma, St. Louis, MO, USA) for 1 h before the 24 h exposure period because the VEGF-stimulated cell proliferation is mediated by increases in the intracellular Ca^2+^ concentration [23].

### ROS measurement

The intracellular ROS production was measured with the fluorescent probe 2′,7′-dichlorofluorescein diacetate (DCFH-DA; Invitrogen A/S Taastrup, Denmark) [24]. A 10 mM DCFH-DA stock solution was made in methanol. 5×10^4^ THP-1a cells or 2×10^4^ HUVECs in a 96-wells plate were stained with 2 µM DCFH-DA in Hanks balanced salt solution (Sigma-Aldrich) at 37°C for 15 min and then rinsed twice in Hanks' balanced salt solution. The THP-1 suspension cells were incubated with probe as described above and then transferred to a 96 well plate as 5×10^4^ cells/well. The cells were then incubated with CB in Hanks' balanced salt solution and the fluorescence was measured every 15 min for 3 h (λ_ex_ = 485 nm; λ_em_ = 538 nm) in a fluorescence spectrophotometer (Fluoroskan Ascent FL; Labsystems). The accumulated ROS production over time was calculated as the area under the curve (AUC). The results are reported as fold increase compared with the control. The ROS production showed a bell-shaped concentration-effect relationship with a peak level at 12.5 µg/ml. This has also been observed in earlier investigations with CB and other types of particles with the same protocol [10], [11], [25], [26]. We show the results from the cell cultures that were exposed to 25 or 100 µg/ml, although these results were not included in the statistical analysis.

As we observed increased lipid accumulation in THP-1a cells after 24 h exposure to lower concentrations of CB (see in results section), we also measured intracellular ROS production in THP-1a cells after exposure to lower concentrations of CB. Here 5×10^4^ THP-1a cells were exposed to CB concentrations of 0.25, 2.5, 25, 250, 2500 ng/ml for 24 h, rinsed, and the ROS production was measured as indicated above.

We also detected the ROS production by fluorescence microscopy. After exposure, 2×10^4^ HUVECs or 5×10^4^ THP-1a on 8-well microscopy chamber slides (Ibidi, Munich, Germany) were stained with 25 µM DCFH-DA in medium for 30 min, rinsed twice with medium, and then examined by combined differential interference contrast (DIC) and fluorescence microscopy in a Leica AF6000 inverted widefield microscope with a 63 times dry objective with NA 0.7 (Leica Microsystems GmbH, Wetzlar, Germany).

### Intracellular GSH concentration

The intracellular GSH concentration was measured with the fluorescent probe ThioGlo-1 (Covalent Technologies, Inc., Walnut Creek, CA, USA) [27]. A 2 mM ThioGlo-1 stock solution was made in DMSO. 5×10^4^ THP-1, 5×10^4^ THP-1a or 2×10^4^ HUVECs seeded in 96-well plates was incubated with CB for 3 h or 24 h. After exposure, the cells were rinsed once with PBS, incubated with 10 µM ThioGlo-1 for approximately 5 min and then measured by fluorescence spectrophotometer (λ_ex_ = 355 nm; λ_em_ = 460 nm). A standard curve was made by incubating 10 µM ThioGlo-1 with GSH ranged from 16 to 0.125 µM (two fold dilution). The GSH concentration is expressed as nmol/10^6^ cells, and is considered to be an observation of GSH equivalents.

### BSO treatment

The intracellular GSH concentration was reduced by incubating the cells with 100 µM of BSO (Sigma-Aldrich, St. Louis, MO, USA) for 24 h. The cells were subsequently rinsed with PBS and exposed to CB or TNF.

### THP-1 cell adhesion assay

The adhesion of THP-1 cells onto HUVCs was performed as previously described [3]. Briefly, 2×10^4^ HUVECs in 96-well plates were co-cultured with 5×10^3^ BrdU-labeled THP-1 cells, and exposed to CB or the positive controls TNF (100 ng/ml) and PMA (100 ng/ml) for 24 h. We assessed the CB-mediated interaction between THP-1 cells and BSO pre-treatment HUVECs in co-culture. Only the HUVECs were pre-treated with 100 µM of BSO for 24 h because there was increased CB-mediated ROS production in BSO pre-treated HUVECs, but not in THP-1 cells.

After exposure, the cells were rinsed twice with 100 µl PBS and the BrdU contents both in the co-culture and supernatant were determined according to manufacturer's instructions (Roche Diagnostics GmbH, Mannheim, Germany) as indicated in Cell proliferating assay section. The percentage of THP-1 cells remaining attached in the co-culture was calculated [3].

### Measurement of ICAM-1 and VCAM-1

The surface expression of ICAM-1 and VCAM-1 was measured with a modified ELISA procedure [10], [13], [26]. The expression of cell adhesion molecules were investigated in cultures with 2×10^4^ HUVECs, 1.8×10^4^ HUVEC +5×10^3^ TPH-1a, 1×10^4^ HUVECs +2.5×10^4^ THP-1a, or 5×10^4^ THP-1a in 96-well plates. The cells were exposed to CB or TNF as positive control (100 ng/ml) for 24 h. Subsequently, the cells were incubated with anti-ICAM-1 or anti-VCAM-1 (both from R&D systems, Abingdon, UK) in a 1∶500 dilution for 1 h at 37°C. After being rinsed for three times in 1% BSA medium, the cells were incubated with 1∶25 000 diluted secondary antibody (anti-goat IgG peroxidase coupled antibody; Sigma, MI, USA) in PBS with 0.1% Tween 20 for 1 h on ice. After washing 5 times with ice-cold PBS/Tween 20 solution, the cells were incubated with a substrate solution containing 0.4 mg/ml o-phenylenediamine (OPD; Sigma, St. Louis, MO, USA) and 3.5 mM H_2_O_2_ in phosphate-citrate buffer (phosphate-citrate buffer tablets, Sigma) for 30 min in the dark. The absorbance was measured in at 450 nm.

We assessed the interference of particles with the assay by exposing HUVECs to 100 ng/ml TNF for 24 h to induce the expression of ICAM-1 and VCAM-1, followed by 30 min exposure to CB. This short CB exposure was expected to have little effect on ICAM-1 and VCAM-1 expression on HUVECs, whereas the adherent CB particles might affect the measurement in the ICAM-1 and VCAM-1 assays.

### Lipid accumulation in THP-1a cells

The lipid accumulation in THP-1a was measured by the fluorescent probe Nile Red (Sigma, St. Louis, MO, USA). THP-1a cells (5×10^4^ cells/well) in 96-well black plates were exposed to various concentrations of CB for 24 h, with or without the presence of 1 mM NAC. After washing, the cells were incubated with either medium containing 1% BSA (for control) or 0.5 mM free fatty acid (FFA; oleic/palmitic acid, 2∶1; in medium with 1% BSA) for another 3 h. After one wash, the cells were stained in Hanks solution containing 0.5 µg/ml Nile red and 0.01% Pluronic F127 (Sigma-Aldrich, St. Louis, MO) for 15 min in the dark and subsequently rinsed twice. The fluorescence was measured in a fluorescence spectrophotometer (λ_ex_ = 544 nm, λ_em_ = 590 nm). There was a remarkable decrease in the Nile red signal at the concentration of 100 µg/ml; probably due to the interactions of CB with the probe. Therefore we show the results from the cell cultures that were exposed to 0 to 25 µg/ml CB.

The lipid accumulation was further assessed with fluorescence microscopy using the two different fluorescent probes Nile Red and Bodipy 493/503 (Molecular Probes, Eugene, OR). Briefly, 5×10^4^ THP-1a cells were seeded on 8-well microscopy chamber slides and after exposure to particles (25 ng/ml or 2.5 µg/ml) and/or FFA, the cells washed with PBS and fixed with 4% para-formaldehyde for 30 min at room temperature. After another wash the fixed cells were stained 10 min with Nile Red (as described above) and Hoechst 33342 or Bodipy (1 µg/ml) and Hoechst 33342 followed by washing and addition of two drops of mounting media (Ibidi). The microscopy was done with a Leica AF6000 inverted wide field microscope with a 40 times dry objective with NA 0.7. Each sample was captured as a z-stack in 5 random areas including at least 50 cells, followed by 3D de-convolution and 3D projection using Leica LAS AF 2.6.0.7266. The resulting images were analyzed with ImageJ (1.47V) to obtain the area of lipid droplets per cell.

### Gene expression of *GCLM*, *HMOX1* and *VCAM1* in HUVECs

The mRNA levels of *GCLM* (Gene ID: 601176), *HMOX1* (Gene ID: 141250) *and VCAM1* (Gene ID: 19225) were measured in HUVECs as previously described [25]. HUVECs (5×10^5^ cells/well) were seeded in 0.1% gelatine pre-coated 6-well plates and exposed to 100 µg/ml of CB for 3 h. Total RNA was extracted using TRIzol reagent (Invitrogen A/S, Taastrup, Denmark) and treated with DNAse (Promega Biotech AB, Denmark). The cDNA synthesis was done with the High Capacity cDNA Reverse Transcription Kit based on the GeneAmp PCR system 2700. The quantitative RT-PCR reactions were carried out with TaqMan Gene Assays on ABI PRISM 7900HT. All kits were obtained from Applied Biosystems, Naerum, Denmark. We used 18S rRNA as reference gene (Eukaryotic 18S rRNA Endogenous Control, 4352930E, Applied Biosystems, Naerum, Denmark). The gene expression levels were reported as the ratio between the mRNA level of the target genes and the 18S rRNA reference gene using the comparative 2^−ΔCt^ method.

### Statistics

The results were analyzed by full-factorial ANOVA with least statistical difference as post-hoc test or linear regression analysis using Statistica version 5.5 (StatSoft Inc., Tulsa, OK, USA). Tests for normal distribution of residuals were carried out with the Kolmogorov-Smirnov test. The distribution of residuals for the ROS production did not follow a normal distribution as assessed by the Kolmogorov-Smirnov test, whereas it was by the Shapiro-Wilk test. The results of BSO pretreatment for CB-mediated ROS production in cells were not normally distributed on normal scale, whereas a log-transformation produced residuals that were normally distributed without affecting the statistically significant differences between the groups. The residuals of the ANOVA analysis of the VCAM-1 expression did not follow a normal distribution on either normal or log-transformed scale. However, this was because of a low value for the positive control (TNF) in one experiment; removal of this result produced residuals that followed a normal distribution. For the assessment of the interaction of BSO pretreatment, the residuals of the VCAM-1 expression was not normally distributed, which was due to one high value in the BSO group that was exposed to 25 µg/ml of CB. Removal of this result from the regression analysis produced residuals with normal distribution, whereas it did not affect the statistical outcome of the test. P–values<0.05 were considered to be statistically significant.

## Results

### Cytotoxicity

**Figure 1:**
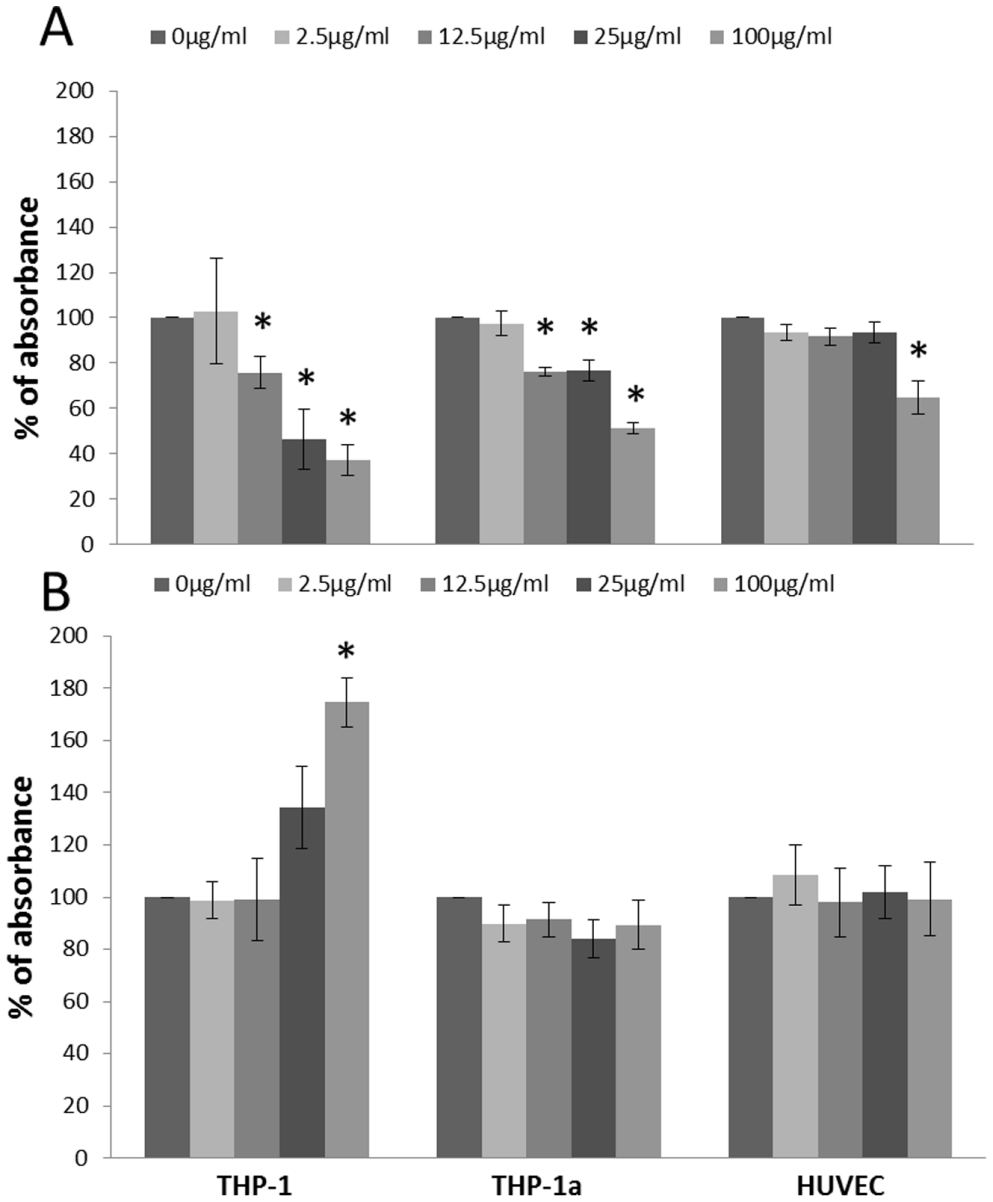
Cytotoxicity measured by the WST-1 assay (A) and LDH leakage (B) after 24 h CB exposure of THP-1, THP-1a and HUVECs. The data represent the percentage of absorbance compared with the unexposed control cells. Bars are means ±SEM of three independent experiments. *P<0.05 compared with unexposed cells.

The results from the WST-1 assay showed increased cytotoxicity in the three types of cells at 100 µg/ml (P<0.05), whereas the exposure to 12.5 and 25 µg/ml of CB also decreased the WST-1 formation THP-1 and THP-1a cells (P<0.05) (Figure 1A). The results from the LDH assay only showed increased membrane leakage at 100 µg/ml CB in THP-1 cells (P<0.05) (Figure 1B).

The viability of THP-1 cells after exposure for 24 h to CB was also measured by the trypan blue assay (Figure S1 in File S1). The viability of both the control and 100 µg/ml CB exposed THP-1 cells were over 90%, and there was no effect of CB exposure.

### BrdU cell proliferation assay

**Figure 2:**
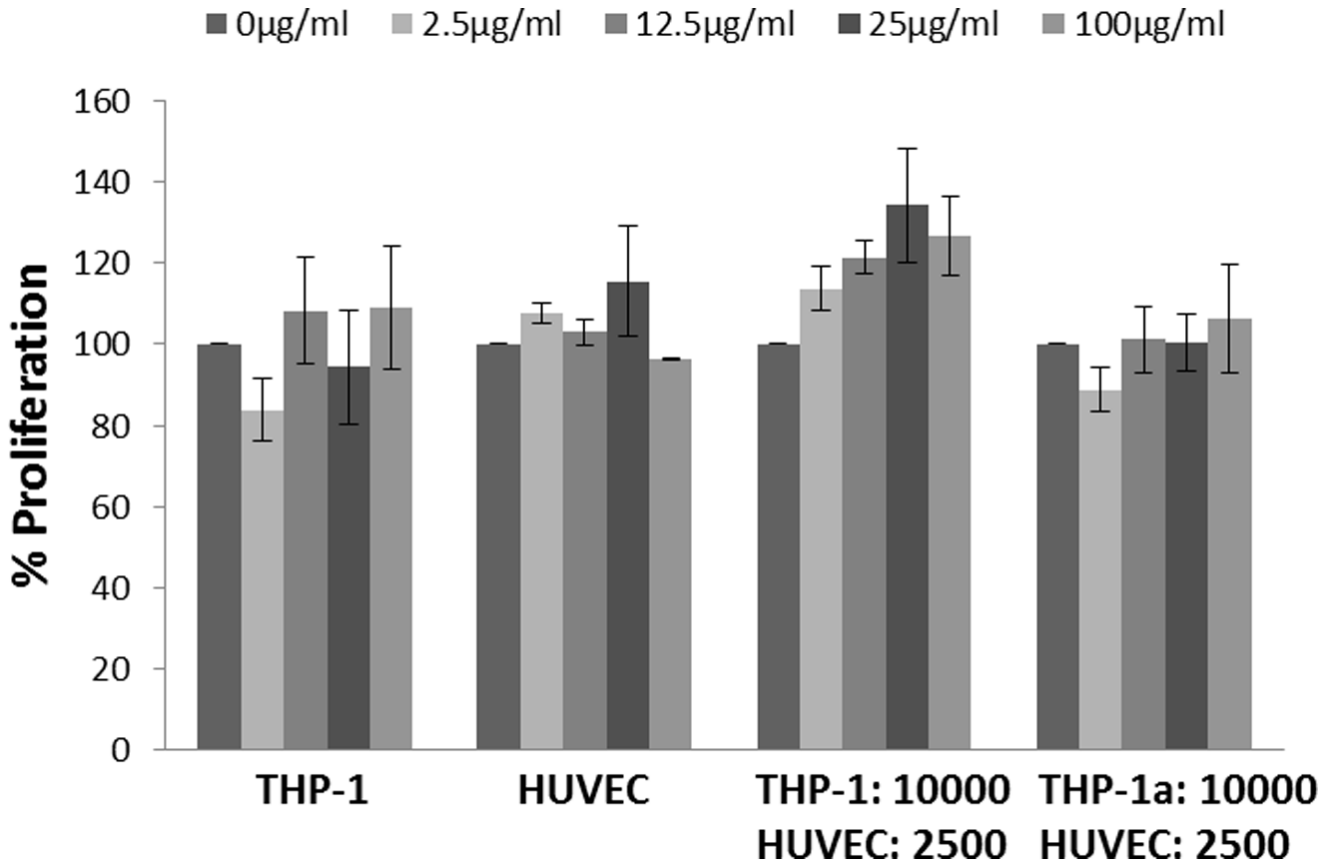
Cell proliferation of THP-1 and HUVECs in monocultures or co-culture after 24 h exposure to CB, measured by the BrdU assay. Data are expressed as percentage of absorbance compared with the unexposed cells. The bars are means ±SEM of at least three independent experiments with n = 3 for each. *P<0.05 compared with unexposed cells.

Figure 2 shows that the proliferation rate of THP-1 cells, HUVECs or co-cultures of both cell types was not significantly affected by the 24 h exposure to CB. The cell proliferation rate was also unaltered after treatment with TNF (83±4%) or VEGF (95±6%), indicating that the HUVECs had maximal proliferation capacity. The effect of pre-treatment of HUVECs with the Ca^2+^ chelator BAPTA-AM, which interferes with the VEGF-mediated increase in intracellular Ca^2+^ concentration, decreased the cell proliferation in HUVECs that were incubated in the presence (26±3%) or absence of VEGF (22±4%).

To confirm the lack of effect on the THP-1 cells, their number was also counted after 24 h CB exposure (Figure S2 in File S1). The results showed that the exposure to 100 µg/ml of CB did not affect the number of THP-1 cells.

### Intracellular GSH concentration

**Figure 3:**
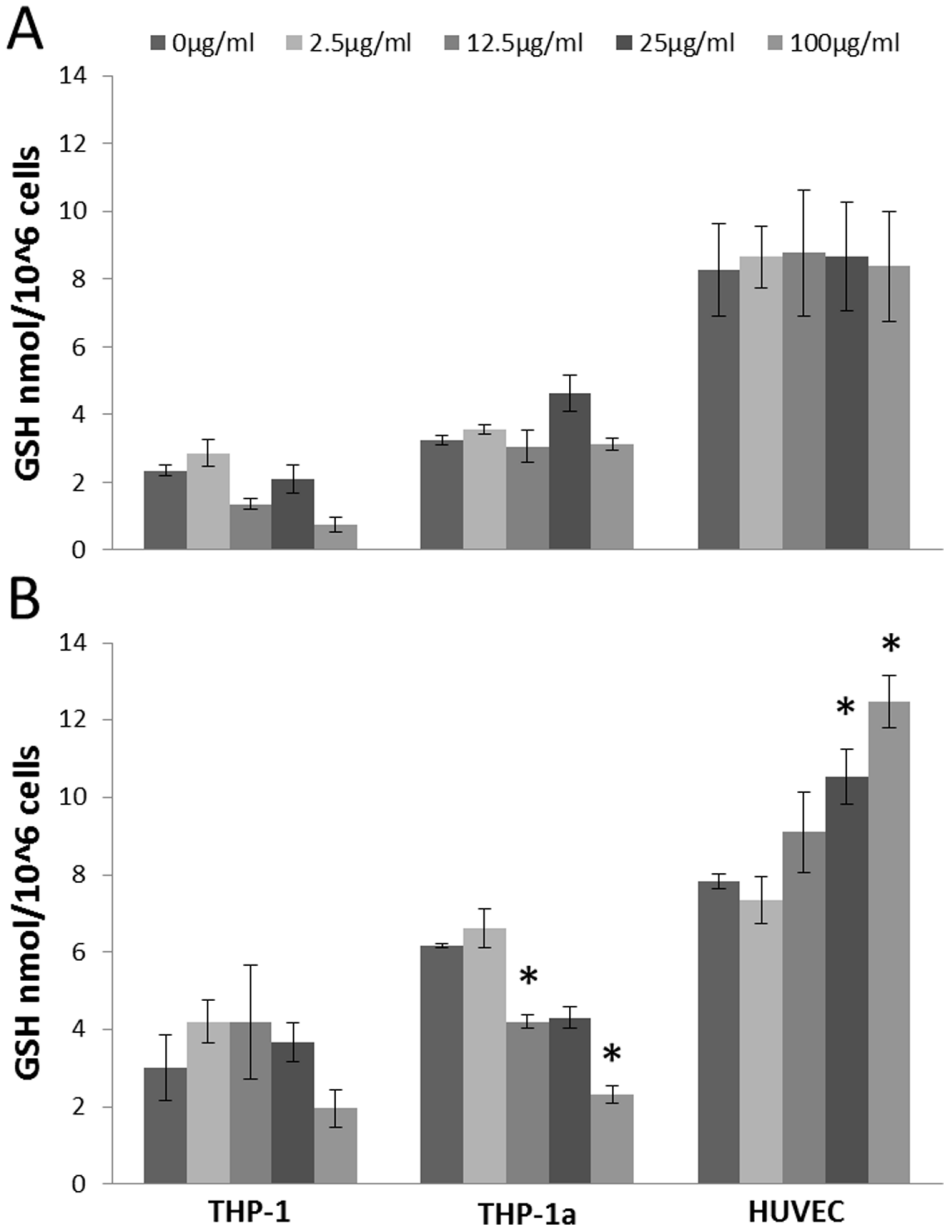
GSH concentration in HUVECs, THP-1 or THP-1a cells after 3 h (A) or 24 h (B) exposure to CB. Data are expressed as nmol/10^6^ cells and bars are means ±SEM of three independent experiments. *P<0.05 compared with unexposed cells.

Figure 3 shows the GSH concentration in cells after exposure to CB. The concentration of GSH was unaltered in all cell types after 3 h exposure to CB (Figure 3A). The GSH concentration was decreased in the THP-1a cells after 24 h exposure to 12.5 (P<0.05), 25 (P = 0.056) and 100 µg/ml CB (P<0.05). In the HUVECs, 24 h exposure to 25 or 100 µg/ml CB (P<0.05) increased the GSH content, whereas there was no difference in the GSH concentration in the CB exposed THP-1 cells. The experiments for the 3 and 24 h exposures were carried out on different days. Therefore, we have not statistically analyzed for differences in the absolute GSH values in the cells after 3 or 24 h incubation. The exposure to PMA, which was used to activate the THP-1 cells, did not affect the GSH concentration in THP-1 cells (data not shown).

### Intracellular ROS production

**Figure 4:**
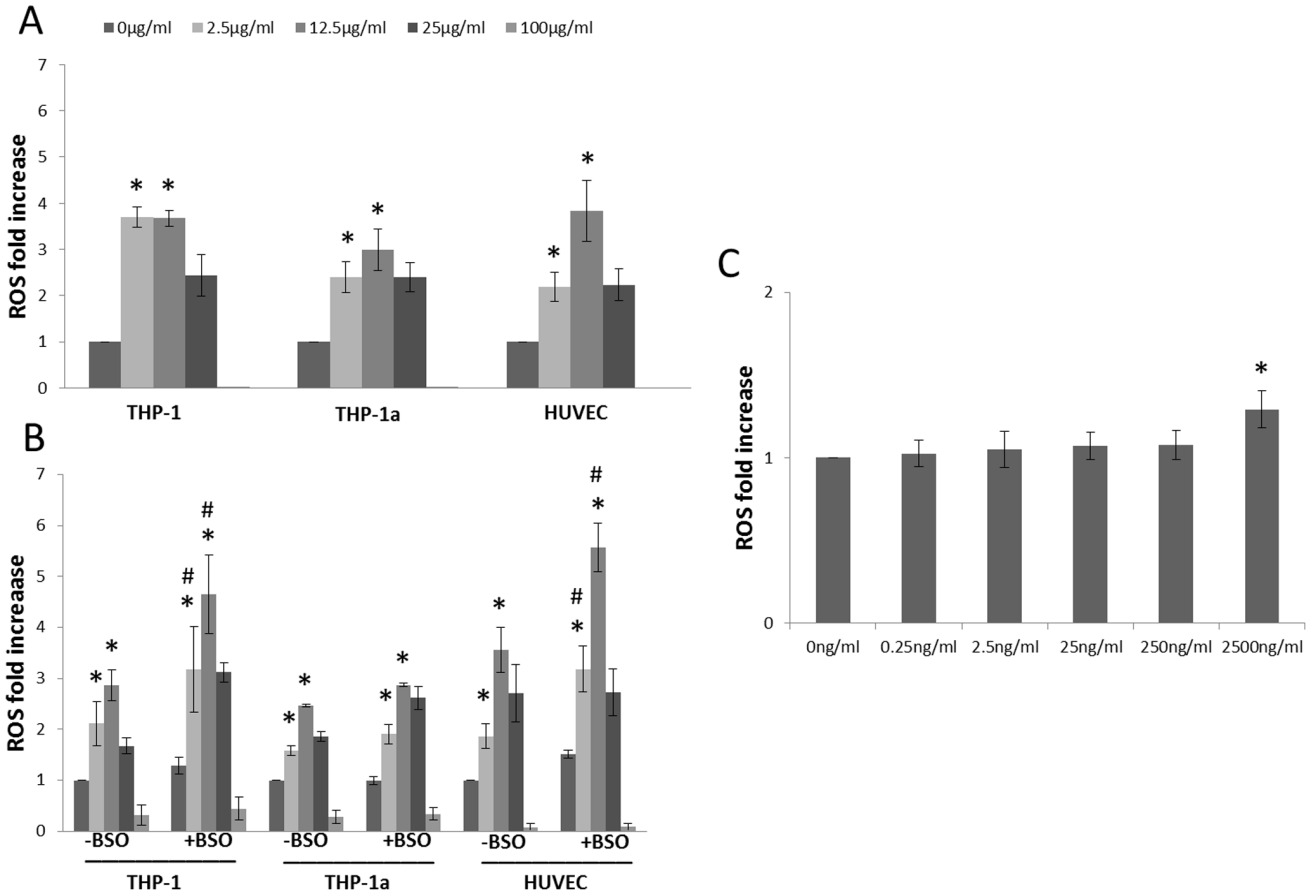
Intracellular ROS production during 3 h incubation with CB in HUVECs, THP-1 or THP-1a cells (A) or cells with or without BSO pre-treatment (B). C. Intracellular ROS production in THP-1a cells after 24 h exposure to lower concentrations of CB. Data are expressed as fold increase of ROS compared with unexposed control cells and bars are means ±SEM of 3–4 independent experiments. *P<0.05 compared with unexposed cells. ^#^P<0.05 compared with BSO-untreated groups.

The 3 h exposure to CB was associated with increased intracellular ROS production in all cell types (Figure 4A). In keeping with earlier findings, we observed bell-shaped concentration-response curves for the intracellular ROS production [10], [13], and the results from 25 and 100 µg/ml were not included in the statistical analysis as these concentrations are speculated to interfere with the measurements [10], [25]. The ROS production was increased at the concentrations of 2.5 and 12.5 µg/ml in the THP-1 (P<0.05), THP-1a cells (P<0.05) and HUVECs (P<0.05). The intracellular ROS generation was further directly observed by fluorescent microscopy to investigate the possible interaction between the particles and the measurement. It showed that the CB exposure induced ROS production in a concentration-dependent manner and there was no obvious decline in ROS signal in HUVECs or THP-1a cells after exposure to 100 µg/ml CB as compared with control or 12.5 µg/ml of CB (Figure S3 in File S1).

The exposure to 10 and 100 ng/ml PMA, which was used to activate the THP-1 cells, increased the ROS production in THP-1 cells by 1.36-fold (95% CI: 0.98–1.72) and 1.57-fold (95% CI: 1.13–1.88), respectively (data not shown).

In another set of experiments we pre-treated the cells with 100 µM of BSO to reduce the intracellular concentration of GSH. The pre-treatment with 100 µM of BSO resulted in a 30% (from 7.2 to 5.0 nmol/10^6^ cells), 28% (from 4.7 to 3.4 nmol/10^6^ cells) decrease and no effect (from 4.2–4.7 nmol/10^6^ cells) on the level of GSH in HUVECs, THP-1a and THP-1 cells, respectively. This pre-treatment was associated with increased CB-induced ROS production in THP-1 cells exposed to 2.5 (P<0.05) or 12.5 µg/ml (P<0.05), whereas the BSO pre-treatment did not affect the CB-induced ROS production in THP-1a cells. The BSO pre-treated HUVECs also showed higher ROS production as compared with non-treated HUVECs after exposure to 2.5 (P<0.05) or 12.5 µg/ml (P<0.05) of CB (Figure 4B).

As we observed increased lipid accumulation in THP-1a cells after 24 h exposure to lower concentrations of CB (see result below), we also measured intracellular ROS production in THP-1a cells after 24 h exposure to lower concentrations of CB to correlate intracellular ROS production with lipid accumulation (Figure 4C). 2500 ng/ml CB exposure significantly increased intracellular ROS production (p<0.05) by 1.29-fold (95% CI: 1.03–1.55 fold), whereas lower concentrations of CB did not significantly affect intracellular ROS production.

### ICAM-1 and VCAM-1 surface expression

**Figure 5:**
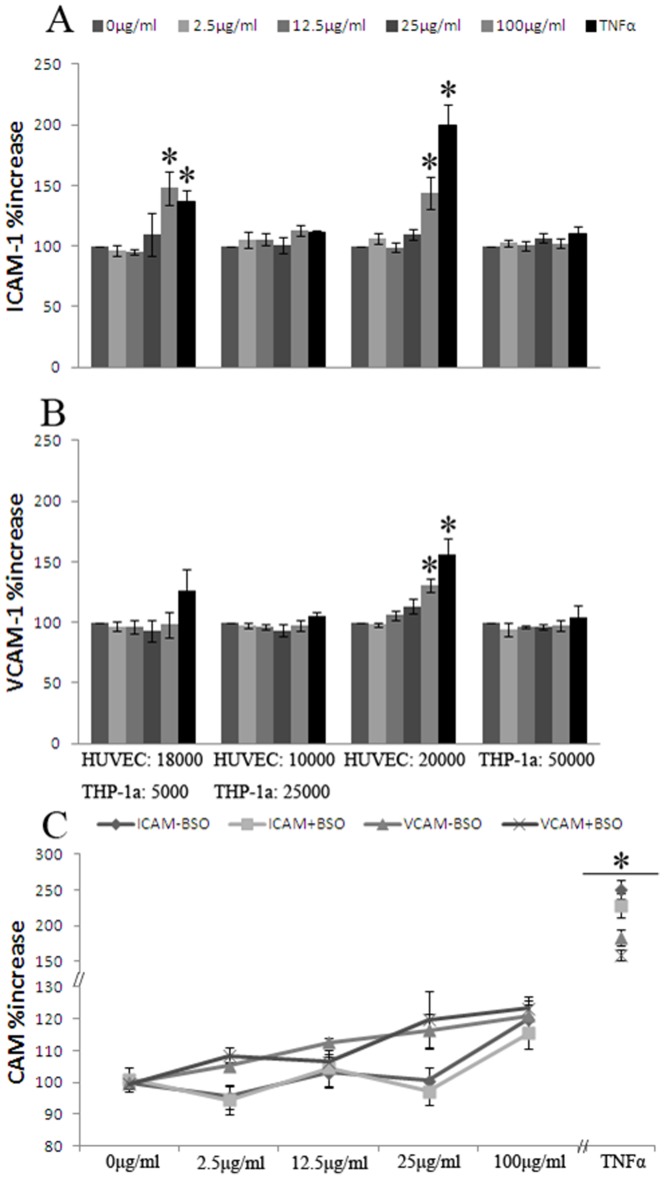
ICAM-1 and VCAM-1 surface expression after 24 h CB exposure in mono-cultures of HUVECs or THP-1a cells, co-cultures with 90% HUVECs and 10% THP-1a cells or 50% HUVECs and 50%THP-1a cells with number of seeded cells indicated (A and B) and effect of BSO pre-treatment on ICAM-1 and VCAM-1 surface expression in HUVECs (C). Data are expressed as percentage of absorbance of unexposed control cells and bars are means ±SEM of at least three independent experiments. *P<0.05 compared with unexposed cells.

Figure 5 shows the ICAM-1 and VCAM-1 surface expression after 24 h of CB exposure in HUVECs and THP-1a monocultures and co-cultures of these. The ICAM-1 expression was significantly increased in 100 µg/ml CB- exposed HUVEC monocultures (P<0.01), the co-culture of 1.8×10^4^ HUVEC +5×10^3^ THP-1a (P<0.01), and the TNF-exposed (positive control) (P<0.001) (Figure 5A). The VCAM-1 expression was only significantly increased after 100 µg/ml of CB or TNF-treatment in HUVEC monocultures (P<0.01) (Figure 5B).

Figure 5C shows the effect of BSO pre-treatment on the CB-mediated expression of ICAM-1 and VCAM-1 in HUVECs. The exposure to CB increased the expression of ICAM-1 (P<0.01) and VCAM-1 (P<0.01), whereas no difference in the slopes of the CB-mediated concentration-response relationships in HUVECs without or with BSO pre-treatment was observed (P = 0.91 for ICAM-1 and P = 0.70 for VCAM-1). The slopes (β±SE) were 0.16±0.07 (P<0.05) and 0.15±0.08 (P = 0.08) for the CB-mediated ICAM-1 expression in HUVECs without or with BSO pre-treatment, respectively. For the CB-mediated VCAM-1 expression in HUVECs, the slopes were 0.18±0.08 (P<0.05) and 0.14±0.05 (P<0.01) without or with BSO pre-treatment, respectively. The reference control TNF also increased the expression of ICAM-1 (P<0.001) and VCAM-1 (P<0.001) in the HUVECs. The effect of TNF was not affected by pretreatment with BSO for ICAM-1 (P = 0.39) and VCAM-1 (P = 0.15).

The possible interaction effect of CB particles on the ICAM-1 and VCAM-1 assays is shown in Figure S4 in File S1. TNF exposure for 24 h increased the ICAM-1 and VCAM-1 expression approximately 3-fold on HUVECs, whereas a subsequent 30 min exposure to CB only caused a slight decrease in the antibody-based measurement of ICAM-1 and VCAM-1, indicating that no interaction between CB and the antibody-based detection of cell adhesion molecules is taking place.

### THP-1 adhesion

**Figure 6:**
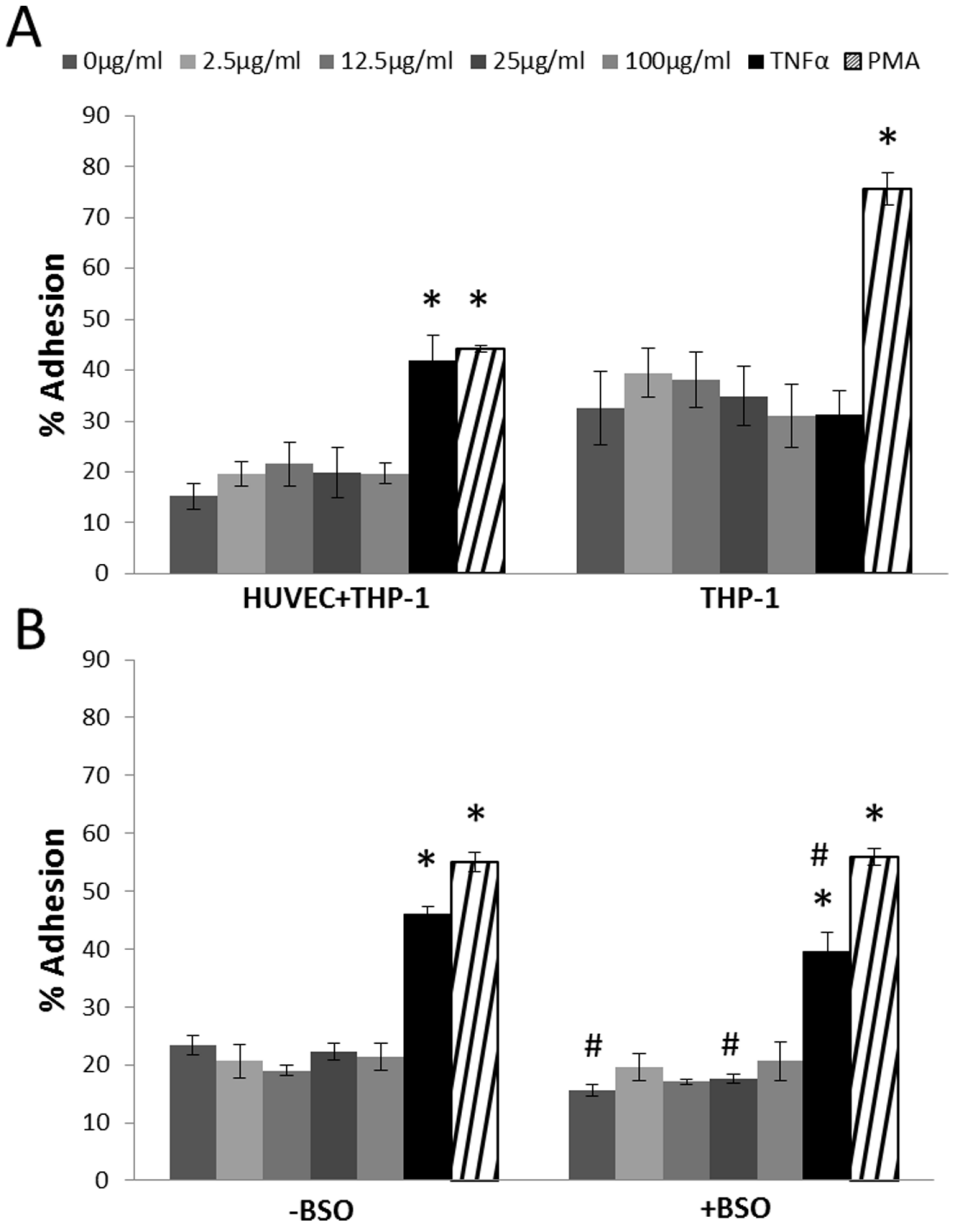
THP-1 adhesion to HUVEC cells or culture dishes assessed by BrdU labeling after 24 h CB exposure (A). In a separate experiment, HUVECs were exposed to 100 µM of BSO for 24 h before they were exposed to CB in co-culture with THP-1 cells (B). Data are expressed as percentage of adherent THP-1 cells compared with the total number of THP-1 cells (cell culture+supernatant) and bars are means ±SEM of three independent experiments *P<0.05 compared with unexposed cells, ^#^P<0.05 compared with co-cultures where the HUVECs were not pre-treated with BSO.

The CB exposure did not alter the level of adhesion of THP-1 cells to the mono-layer of HUVECs or culture dishes (Figure 6A). The positive control TNF was associated with increased adhesion of THP-1 cells onto HUVECs (P<0.05) and PMA (positive inducer of THP-1a macrophages) significantly increased THP-1 adhesion to both HUVECs (P<0.05) and culture dishes (P<0.05).

The pre-treatment of HUVECs with BSO was associated with decreased adhesion of THP-1 cells onto HUVECs in co-cultures that were not exposed to CB (P<0.05) and those that were exposed to TNF (P<0.05). Although we observed a similar tendency in co-cultures exposed to 25 µg/ml of CB (P<0.05), it was not a concentration-dependent effect (Figure 6B).

### Lipid accumulation in THP-1a cells

**Figure 7:**
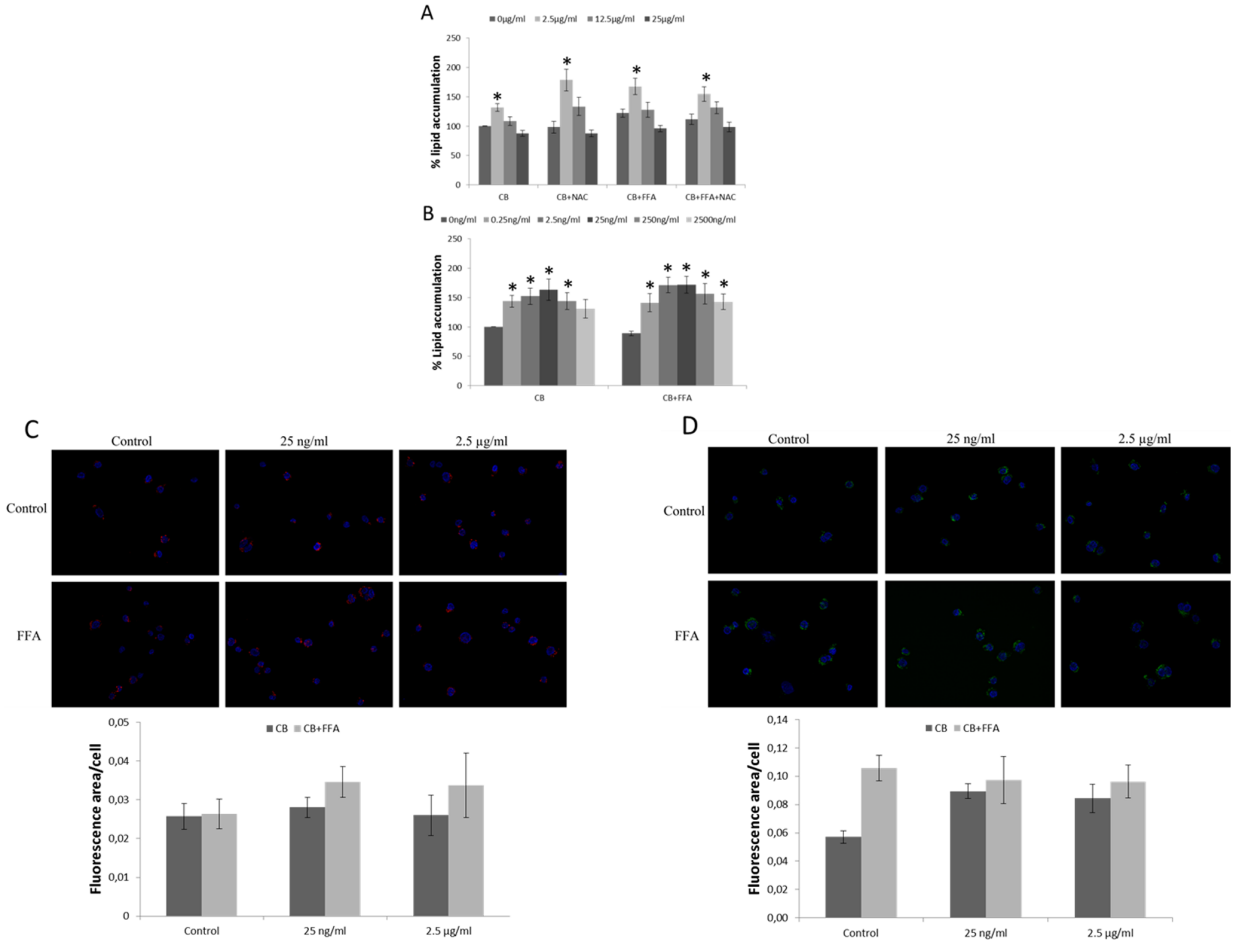
Accumulation of lipids in THP-1a cells after 24 h exposure to CB with or without the presence of NAC (A) or lower concentrations of CB (B) and subsequently treated with free fatty acids (FFA) for 3 h. Data are expressed as percentage increase of lipid accumulation compared with unexposed control cells and bars are means ±SEM of 4–10 independent experiments. *P<0.05 compared with unexposed cells. Semi-quantitative determination of lipid accumulation were performed by microscopy with Nile Red and Bodipy 493/503: C) Cells are stained with Nile Red (red) and Hoechst (blue), D) Cells are stained with Bodipy 493/503 (green) and Hoechst (blue) and representative images are shown in top, and calculation of the lipid fluorescence area per cell shown below calculated from 5 independent areas with SEM.

The result of the lipid accumulation in THP-1a cells is shown in Figure 7. Pre-exposure to CB for 24 h increased lipid accumulation in THP-1a cells with a bell-shaped concentration-response curve. The CB exposure was associated with a significant increase in lipid accumulation at a concentration of 2.5 µg/ml (p<0.05). There was 30% (95% CI: 6.9%–60%) and 36% (95% CI: 11%–67%) higher lipid load in the cells that had been exposed to 2.5 µg/ml of CB and co-treated without or with FAA, respectively. In the cells that had been exposed to 2.5 µg/ml of CB and NAC, there was 82% (95% CI: 45%–123%) and 38% (95% CI: 10%–74%) higher lipid load in cells that were co-treated without or with FFA, respectively. The 3 h exposure to FFA was associated with a minor 11% (95% CI: 3.0%–20%) increase of the lipid level (p<0.05 for single-factor effect of FFA treatment) (Figure 7A). Pre-exposure to lower concentrations of CB ranging from 2500 ng/ml to 0.25 ng/ml for 24 h also increased lipid accumulation in THP-1a cells with or without further exposure to FFA (p<0.05 for 2500 ng/ml and p<0.01 for other concentrations) (Figure 7B).

Using fluorescence microscopy for further semi-quantitative determination of lipid accumulation, we observed increased lipid accumulation in THP-1a cells that were incubated with CB and/or FFA. Staining with Nile Red showed predominantly increased lipid content in cells exposed to CB and FFA (Figure 7C), whereas Bodipy 493/503 staining revealed increased lipid content in CB exposed cells without FFA treatment (Figure 7D). Although the lipid stains differ somewhat in regard to effect of FFA treatment, the collective interpretation from the fluorescence microscopy is that CB exposure is associated with increased lipid content in THP-1a cells.

### Gene expressions

**Figure 8:**
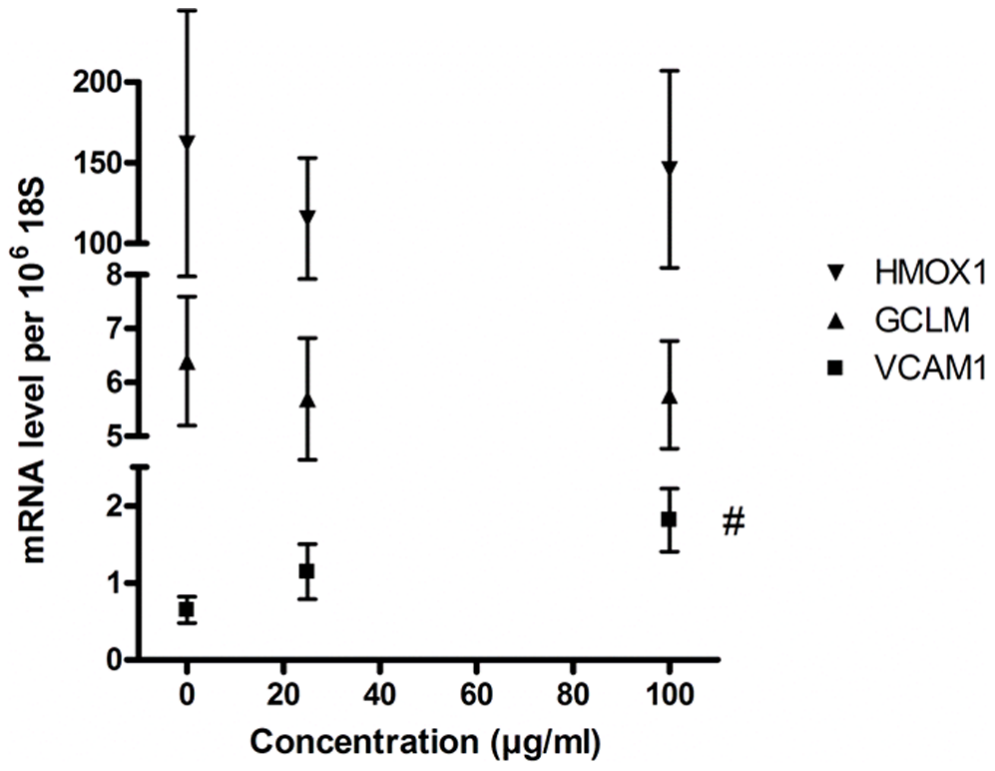
mRNA expression of *GCLM*, *HMOX1* and *VCAM1* in HUVECs after exposure to CB for 3 h. The symbols represent the mean and SEM of three independent experiments. ^#^P<0.05 (linear regression analysis).

Because we observed increased ROS production at 3 h after the CB exposure and increased GSH content in HUVECs at 24 h, we measured the expression of *GCLM*, *HMOX1* and *VCAM1* after 3 h exposure to CB. The results showed that CB exposure did not alter the expressions of *GCLM* (P = 0.75) and *HMOX1* (P = 0.97) in HUVECs, whereas there was a concentration- dependent increase in *VCAM1* expression (P<0.05) (Figure 8).

## Discussion

In this study we assessed the in vitro effects of CB exposure on oxidative stress, endothelial activation and foam cell formation which are important events in the early development of atherosclerosis [2]. The results showed that CB exposure increased the expression of ICAM-1 at protein level and VCAM-1 at both protein and gene level in HUVECs, whereas the adhesion of THP-1cells to HUVECs or culture dishes was unaffected by the exposure. In a previous study, we showed that exposure to particles from combustion of diesel or wood was associated with increased VCAM-1 expression, whereas only the wood smoke particles enhanced the adhesion of THP-1 cells onto HUVECs [3]. It is therefore possible that particle-induced monocyte adhesion onto endothelium depends on the activation of both types of cells. Nevertheless, CB exposure has been found to be associated with vasomotor dysfunction in aorta rings and altered pressure-diameter relationship in mesenteric artery segments from mice [13]. These effects correlated with the observations in HUVECs in mono-culture, including increased expression of cell adhesion molecules and ROS production. However, it can be argued that HUVECs mono-culture is a rather simple exposure system and therefore co-culture was used here. This showed that the addition of THP-1a cells did not increase the CB-mediated expression of ICAM-1 and VCAM-1 on HUVECs. This is in accordance with earlier observations that THP-1 cells did not increase the expression of IL-1β, IL-6, IL-8 and TNF after exposure to CB [28]. Overall, the results indicate that CB possesses the ability to cause a modest activation of endothelial cells *in vitro*.

The CB exposure significantly increased the lipid accumulation in THP-1a cells, suggesting that CB exposure may promote the transformation of monocytes to foam cells, which is an important event in the atherogenesis [2]. This is consistent with previous studies showing that NP exposure could induce intracellular lipid accumulation [6], [7], [11] and it supports the observation that pulmonary exposure to CB accelerated progression of atherosclerosis [16]. However, the presence of the antioxidant NAC showed no effect on lipid accumulation induced by CB, and the concentrations of CB needed to induce lipid accumulation were apparently lower than the concentrations to promote intracellular ROS production in THP-1a cells, which indicated that CB induced lipid accumulation is independent of intracellular ROS production. This is in contrast to the effect of other NPs like cadmium telluride that can induce lipid droplet in an oxidant-dependent way [11].

We measured the intracellular ROS production by the DCHF assay that has been widely used in research on oxidative stress for years [29]. In keeping with earlier findings, we observed bell-shaped concentration-response curves for the intracellular ROS production in experiments were the cells loaded with DFCH before the exposure to CB [10], [13], [25]. This exposure condition enables the possibility to measure the intracellular ROS production in real-time, although the limitation is possible quenching of the signal by the particles in the cell culture medium. We have previously shown that addition of DCFH to HepG2 cells after the exposure to CB was associated with increased intracellular ROS production that did not show a bell-shaped concentration-response curve [30]. Those results showed essentially the same trend as the results in Figure S3 in File S1, albeit it was a different cell type. The collective interpretation of the Printex 90 exposure in our experiments is mediates the generation of intracellular ROS as measured by the DCFH assay. This is supported by studies on other types of probes for intracellular ROS production such as hydroethidium for detection of superoxide anion radicals [31].

It is generally acknowledged that the DCFH assay does not have specificity to particular types of ROS in the context of an intracellular environment. Interestingly some studies have shown that lysosomal permeabilization and subsequent increased release of iron to the cytosol is an important source of intracellular ROS as detected by the DCFH assay [32], [33]. Studies in acellular conditions have also highlighted iron or heme-compounds as potent oxidants of DCFH [34], [35]. It is therefore possible that the increased intracellular ROS production in CB-exposed cells arises as a consequence of “free” iron from compartments such as the lysosome or mitochondria. We have shown in several studies that CB in acellular conditions oxidizes DCFH [12], [13]. In addition, the same type of CB generated ROS in acellular conditions be electron spin resonance [36], indicating the intrinsic ability of CB to generate ROS.

For all cell types there was CB-induced ROS production, whereas the intracellular GSH concentration remained unaffected during the same time period of 3 h CB exposure. To further explore the possible relationship between CB-induced endothelial activation and intracellular ROS level, we reduced the intracellular GSH concentration by BSO pretreatment prior to CB exposure. This increased the CB-induced ROS production especially in the THP-1 cells and HUVECs. The combined results from the BSO pre-treatment experiments indicate that the response is cell type specific, which might be related to the *de novo* synthesis of GSH and its interaction with other cellular components of the antioxidant defense system. We did not attempt to completely deplete the GSH because it might render the cells more susceptible to cytotoxicity by exposure to particles [37]. Too low GSH levels may also affect ROS-mediated redox signaling in macrophages, as seen in another study [38]. The BSO treatment did not affect CB-induced ICAM-1 and VCAM-1 surface expression or the THP-1 adhesion to HUVECs, which indicates that endothelial activation by CB exposure could be independent of oxidative stress. This is consistent with earlier observation where maintenance of ascorbate levels in HUVECs attenuated the CB-induced ROS production without affecting the expression of ICAM-1 and VCAM-1 [10]. Indeed, our results showed that depletion of GSH by BSO pre-treatment resulted in slightly reduced ICAM-1 and VCAM-1 expressions by TNF exposure and significantly reduced the TNF-mediated adhesion of THP-1 cells onto HUVECs. This is consistent with the observation that excessive BSO pre-treatment can lead to cellular adaptive response through the activation of the NRF-2 pathway and thus altered cell survival response [39]. However, the CB exposure for 3 h did not change the expressions of *GCLM* and *HMOX1*. Up regulation of *GCLM* with possibly increased synthesis capacity might be related to the elevated GSH levels found after CB exposure in HUVECs. However, the unaltered *HMOX1* expression is consistent with no sign of oxidative stress and even elevated GSH levels despite ROS formation detected by DCFH in HUVECs. In contrast, it has been shown that the *VCAM1* expression was sensitive to fluctuations in the redox state and mediators that are generated by inflammation [40], whereas the expression of VCAM-1 did not depend on the superoxide anion radical levels in vessel segments form mice [41]. Indeed, we observed an increased expression of *VCAM1* after the 3 h exposure to CB. This indicates that the gene expression of *VCAM-1* might be sensitive to intracellular generation of ROS without overt oxidative stress, or that the mRNA up regulation is unrelated to ROS formation like the enhanced expression of VCAM-1 on the plasma membrane of HUVECs induced by CB.

PMA was used to differentiate THP-1 cells to become adherent macrophage-like cells (THP-1a cells). It has been suggested that ROS have an initial signaling role for the differentiation of THP-1 cells to macrophages [19]. Here we also found that PMA treatment slightly increased ROS production in THP-1 cells, although this was much lower than the CB-induced ROS production. However, the slightly increased ROS production of THP-1a cells as compared with undifferentiated THP-1cells could also be related to higher phagocytosis activity in the former as also evidenced by higher mitochondrial activity and oxidative respiration [42]. The CB induced ROS production was not associated with enhanced adhesion of THP-1 cells to HUVECs or culture dishes.

We measured cell viability by several methods to determine concentrations with minimum cytotoxicity in order to be able to interpret the data related to atherosclerosis processes. The WST-1 and LDH assays are considered to be indicators of effect to mitochondria and cell membrane, respectively, whereas the proliferation ability can be regarded as an overall indicator of the cellular well-being in as much as the cells will not proliferate if they are damaged. The exposure to CB at high concentration was associated with cytotoxicity in THP-1 cells, without any effect on the proliferation in the same exposure period or decreased viability assessed by trypan blue exclusion. This is in agreement with earlier observations that the LDH release was increased in A549 lung epithelial cells after exposure to diesel exhaust particles, although it had little effect on the ability to proliferate and form colonies [43].

There were increased oxidative stress endpoints and lipid accumulation at the concentration of 2.5 µg/ml, whereas the expression of ICAM-1 and VCAM-1 was increased at 25 and 100 µg/ml. These are high but typical concentrations in particle toxicology. The relevance to the human exposure situation is difficult to assess firmly because studies on translocation usually use other types of particles, which can be traced systemically. In perspective, the dose was 1 mg/mouse by i.t. instillation in a study that showed increased level of atherosclerosis after exposure to CB [16]. This corresponds to 10 µg/ml in blood, assuming 1% translocation of the deposited dose in a blood volume of 1 ml in mice. It has further been estimated that a 24 h exposure to1 mg/m^3^ of NPs with a diameter around 14–20 nm would correspond to an average exposure of 0.15 µg/cm^2^ lung surface, which equals 0.24 µg/ml (240 ng/ml) in 96-wells plates [44]. This corresponds to a concentration of 2.4 ng/ml in blood, assuming the same 1% translocation to 1 ml blood. As the Occupational Exposure Limit for carbon black is 3.5 mg/m^3^, the exposure concentration levels used in this study were high and endothelial cells and monocytes will probably never be exposed to such concentrations *in vivo*. Nevertheless, the mechanistic effects have to be investigated both in realistic concentrations as well as under circumstances where effects are observable. On the other hand, we found an increase in the lipid accumulation by CB at concentrations ranging from 2.5 µg/ml to 0.25 ng/ml, which could be achieved *in vivo*. As in atherosclerosis, there are increased level of differentiated macrophages adherent to the endothelium [2], CB exposure could promote the lipid laden foam cell formation *in vivo*.

In conclusion, the results showed that exposure to nano-sized CB increased the expression of ICAM-1 and VCAM-1 on endothelial cells. This expression was not enhanced by BSO pre-treatment, although BSO augmented the CB-mediated ROS generation. In addition, CB exposure increased the lipid accumulation in THP-1 macrophages, but the presence of antioxidants did not affect the lipid accumulation induced by CB. Further, very low concentrations of CB promoted lipid accumulation without obvious effect on intracellular ROS production. Our results suggest that CB induces endothelial adhesion molecule expression and foam cell formation independent of CB-induced ROS generation.

## Supporting Information

File S1 **Figure S1**. Cytotoxicity of CB on THP-1 cells after 24 h exposure as measured by trypan blue assay. Figure S2. Cell number of THP-1 cells after 24 h CB. Figure S3. Representative micrographs showing the intracellular ROS production after 3 h incubation with CB in HUVECs or THP-1a cells. Figure S4. Interaction effect of a 30 min CB exposure on the ICAM-1 and VCAM-1 assays.
(DOC)
