# Supplementary material for: Carbon Black Nanoparticles Promote Endothelial Activation and Lipid Accumulation in Macrophages Independently of Intracellular ROS Production
Source: PLoS One. 2014 Sep 3;9(9):e106711. doi: 10.1371/journal.pone.0106711 (PMC4153655; doi:10.1371/journal.pone.0106711)
Supplement: File S1 — Figure S1. Cytotoxicity of CB on THP-1 cells after 24 h exposure as measured by trypan blue assay. Figure S2. Cell number of THP-1 cells after 24 h CB. Figure S3. Representative micrographs showing the intracellular ROS production after 3 h incubation with CB in HUVECs or THP-1a cells. Figure S4. Interaction effect of a 30 min CB exposure on the ICAM-1 and VCAM-1 assays. (DOC) [file pone.0106711.s001.doc]

**Supporting Information S1**

**Carbon black nanoparticles promote endothelial activation and lipid accumulation in macrophages independently of intracellular ROS production**

Yi Cao, Martin Roursgaard***, Pernille Høgh Danielsen, Peter Møller, Steffen Loft

Section of Environmental Health, Department of Public Health, University of Copenhagen, Denmark.

**To whom correspondence should be addressed:*

Department of Public Health, Section of Environmental Health, University of Copenhagen, Øster Farimagsgade 5, 1014 Copenhagen K, Denmark.

Tel: +45 35327989 ; Fax: +45 32327748; E-mail: mwro@sund.ku.dk.

**Running title**: Endothelial effect and carbon black exposure

Supplemental Figures:

Supplemental Figure S1. Cytotoxicity of CB on THP-1 cells after 24 h exposure as measured by trypan blue assay.

Supplemental Figure S2. Cell number of THP-1 cells after 24 h CB.

Supplemental Figure S3. Representative micrographs showing the intracellular ROS production after 3 h incubation with CB in HUVECs or THP-1a cells.

Supplemental Figure S4. Interaction effect of a 30 min CB exposure on the ICAM-1 and VCAM-1 assays.

Figure S1.


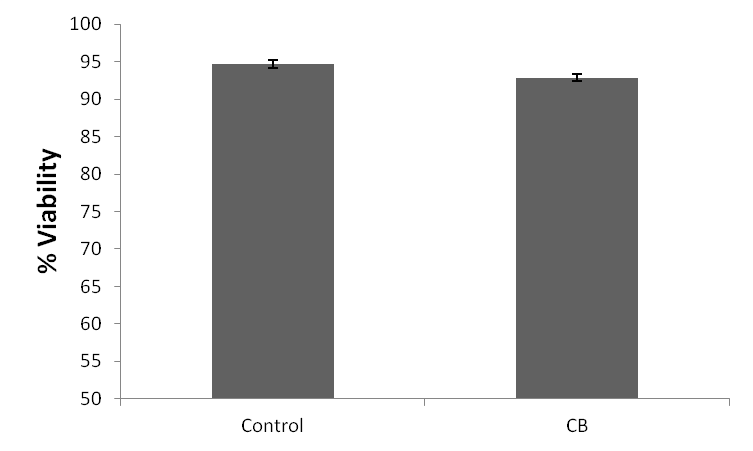


Figure S1. Cytotoxicity of CB on THP-1 cells after 24 h exposure as measured by trypan blue assay. Data are expressed as percentage of living cells (not colored cells) of the total and bars are means ± SEM of three independent experiments

Figure S2.


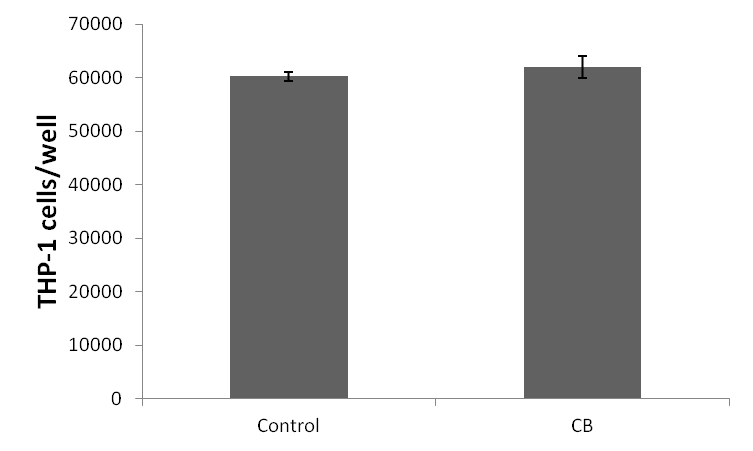


Figure S2. Cell number of THP-1 cells after 24 h CB. Data are expressed as number of THP-1 cells/well bars are means ± SEM of three independent experiments (n=3 for each).

Figure S3.


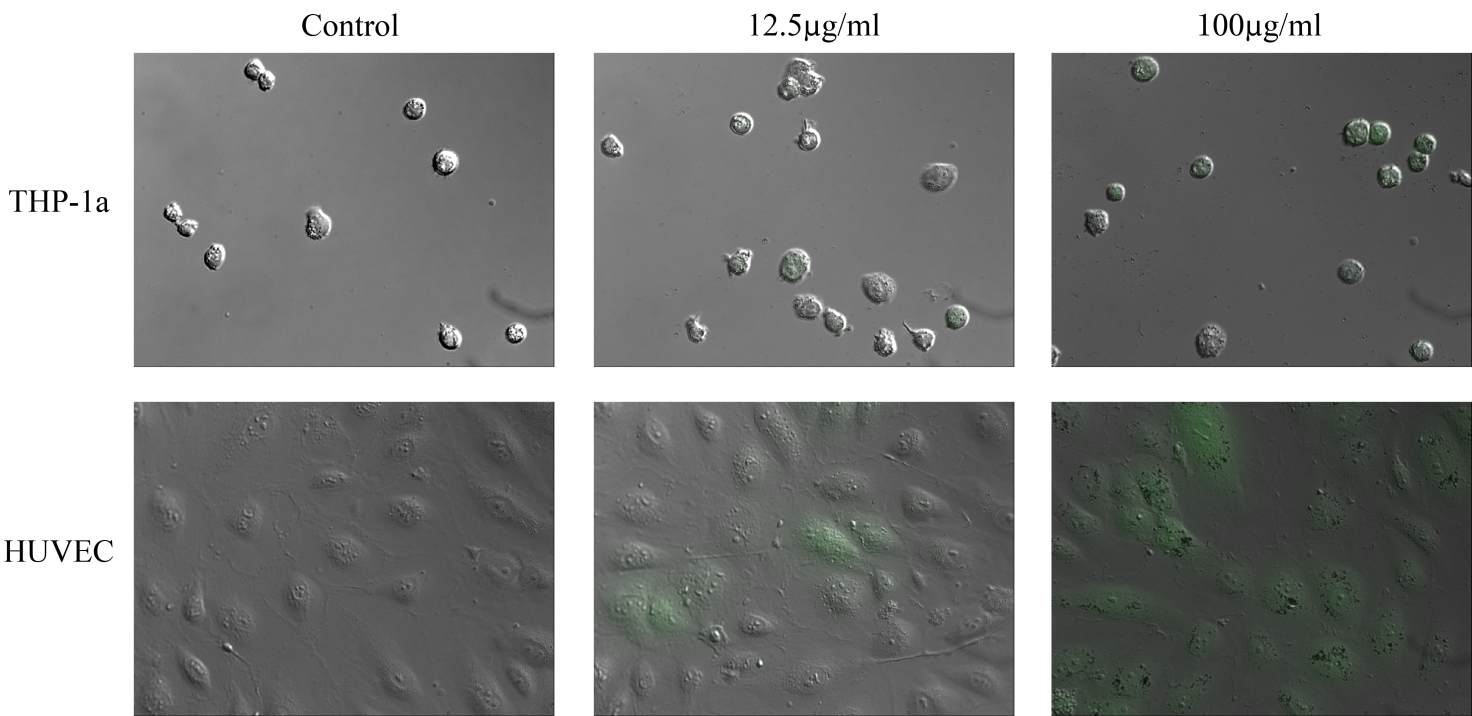


Figure S3. Representative micrographs showing the intracellular ROS production after 3 h incubation with CB in HUVECs or THP-1a cells. The micrographs show an overlay of a DIC image and a fluorescence image showing the activated DCFH probe in green.

Figure S4.


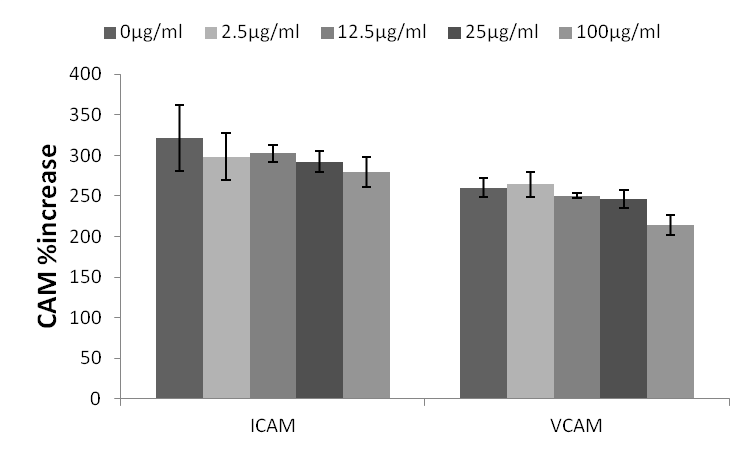


Figure S4. Interaction effect of a 30 min CB exposure on the ICAM-1 and VCAM-1 assays. Data are expressed as percentage of absorbance in cells treated with TNF for 24 h in relation to unexposed control cells and bars are means ± SEM of three independent experiments
